# Prof Miles Padgett (OBE, FRS) from blue-sky research to real-world applications and challenges

**DOI:** 10.1038/s41377-025-01771-8

**Published:** 2025-04-07

**Authors:** Ruidong Xia, Ying Hu

**Affiliations:** 1 Key Laboratory for Organic Electronics & Information Displays (KLOEID), Jiangsu-Singapore Joint Research Center for Organic/Bio Electronics & Information Displays, Institute of Advanced Materials (IAM), Nanjing University of Posts and Telecommunications, 9 Wenyuan Road, Nanjing, 210023 China; 2 Executive Management College of CHN ENERGY, No. 7 Binhe Avenue, North District of Future Science City, Changping District, Beijing, 102211 China; 3 Key Laboratory of Terahertz Optoelectronics, Ministry of Education, Capital Normal University, 105, West Third Ring Road, Haidian District, Beijing, 100089 China

**Keywords:** Applied optics, Lasers, LEDs and light sources, Optical physics

## Abstract

Orbital angular momentum (OAM) research has evolved from a theoretical concept to a tool with diverse applications. Early advancements distinguished OAM from spin angular momentum (SAM), leading to practical innovations such as optical tweezers and quantum entanglement. Compared with SAM, OAM can carry more information, which makes it invaluable for high-capacity data transmission and secure communications. Professor Miles Padgett, a leading scientist in the field of optical momentum, is well-known for his contributions, including the realization of an optical spanner for spinning micron-sized objects, the use of orbital angular momentum to increase the data capacity for communication systems, and the development of an angular form of the Einstein‒Podolky‒Rosen (EPR) quantum paradox. In an enlightening conversation with Light: Science & Applications, he highlighted the fundamental properties of the angular momentum of light, the invention of optical tweezers and optical spanners, and the demonstration of OAM states for extending the alphabet of optical communication using both classical and quantum light. In particular, he explained the various aspects of OAM distinguished from SAM. This interview further explored his collaboration with industry partners that bridges the gap between academic research and real-world applications by using his skill in light shaping in various areas, including his current role as the principal investigator for QuantIC and his group’s work on building novel endoscopes that are the size of the width of a human hair.

As an academic administrator, during his 5-year term as Vice-Principal for Research at the University of Glasgow (2014–2019), Professor Miles Padgett’s efforts led to an improvement in the quality of the University’s research publications from the lower quartile to the upper quartile in the Russell Group of the UKs leading universities. In this interview, he shared his approach to improve research culture to build up research collaboration, secure external funding for conducting cutting-edge research, and translate blue-sky research into real-world impact. In addition to his research success, Miles also serves many important roles for research societies and funding agencies. For example, as the Interim Executive Chair for EPSRC in 2023, his tenure successfully led to a nearly 50% increase in the number of funded Centres for Doctoral Training, corresponding to an additional intake of 1500 students. When asked about his motivation to serve on research committees, he expressed his ambition to shape the direction of science, advocating for areas of science with the potential to impact society. For young scientists, his advice is to understand that perseverance and adaptability are crucial for research career progress while remembering that luck also plays a role—sometime you just have to hang on in.

**Figure:**
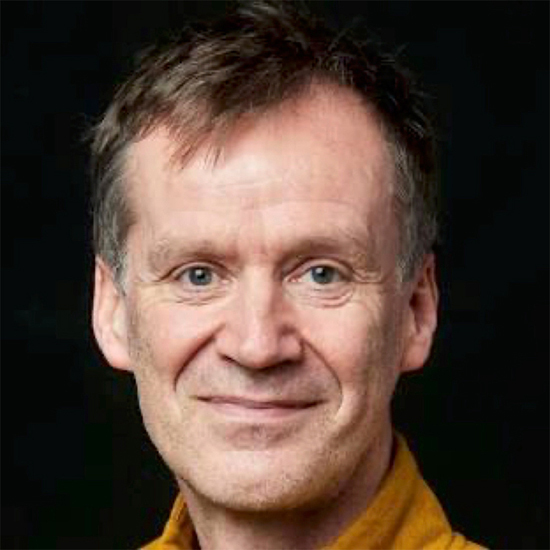
Prof Miles Padgett

**Biography:** Prof. Miles Padgett (OBE FRS) was born on 1st June 1963. He was educated at the University of Manchester, the University of York for a BSc, the University of St Andrews for a MSc, and Trinity College, Cambridge, where he completed his thesis on *Techniques for Ultra-High-Resolution Saturation Spectroscopy and Laser Stabilization in the 10* *μm Spectral Region* and was awarded a PhD in 1988. He is a Royal Society Research Professor of Optics in the School of Physics and Astronomy at the University of Glasgow. He has held the Kelvin Chair of Natural Philosophy since 2011 and served as the Vice-Principal for Research at Glasgow from 2014 to 2019. In 2023, he served as the Interim Executive Chair for EPSRC. From 2014 to 2024 Miles served as Principal Investigator for QuantIC, the UK’s Centre of Excellence for Research, Development, and Innovation in Quantum-Enhanced Imaging. Miles is internationally recognized for his leadership in the field of optical momentum. Miles’s research interests cover all things optical, from the basic ways in which light behaves as it pushes and twists the world around us to the application of new optical techniques for imaging and sensing. This includes utilizing the classical and quantum properties of light to explore the following: the laws of quantum physics in accelerating frames, microscopes that can be used to see through noise, shaped light for overcoming diffraction-limited resolution, and endoscopes that are the size of the width of a human hair. His best-known contributions include the creation of an optical spanner for spinning micron-sized objects, the use of orbital angular momentum to increase the data capacity for communication systems, and the advancement of an angular form of the Einstein‒Podolky‒Rosen (EPR) quantum paradox. Miles was elected a Fellow of the Royal Society of Edinburgh (FRSE) in 2001, a Fellow of Optica in 2011, a Fellow of SPIE in 2012, and a Fellow of the Royal Society (FRS) in 2014. He has been awarded various national and international prizes, such as the Young Medal for his pioneering work on optical angular momentum in 2009, the Royal Society of Edinburgh’s Lord Kelvin Medal in 2014, the Science of Light Prize from the European Physical Society in 2015, the Max Born Award of the Optical Society (OSA) in 2017, the Rumford Medal of the Royal Society in 2019, and the Quantum Electronics and Optics Prize of the European Physical Society in 2021. Since 2019, he has been identified by the Web of Science as a globally highly cited researcher.

**Q1. Could we start with the fascinating field of research you and your colleagues pioneered decades ago: the orbital angular momentum of light? Could you please briefly introduce this work to our readers who may not be familiar with this area?**

The concept of the angular momentum of light, while not entirely new, has gained more attention and a deeper understanding in recent years. That light carries a linear momentum is well-known to all. Interestingly, ALL waves (e.g., light and sound) carry momentum, and for ALL waves, this momentum is equal to the energy in the wave divided by its phase velocity. Light can also carry angular momentum, which comes in two forms: spin angular momentum (SAM) and orbital angular momentum (OAM). Spin angular momentum is associated with the polarization of light. Circularly polarized light, for instance, has a spin angular momentum per photon that is equal to ±ℏ (where ℏ is the reduced Planck constant), corresponding to right or left circular polarization. Actually, this form of angular momentum is hard to understand intuitively but is, of course, essential for understanding atomic transitions and the conservation of angular momentum in the emission/absorption process.

**Figure:**
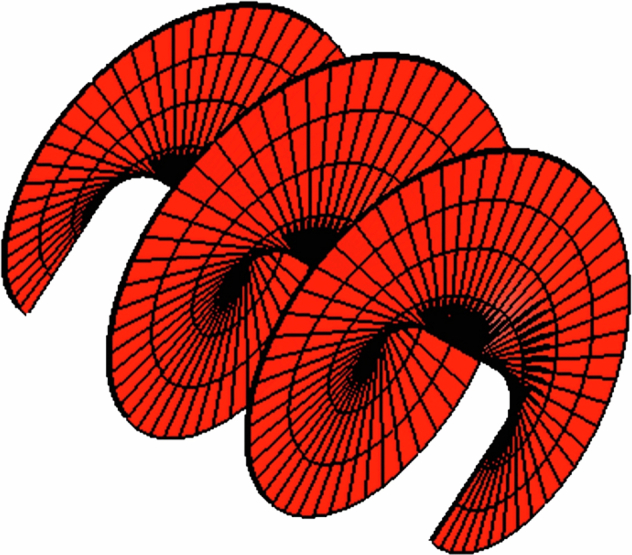
Scheme showing OAM

Whereas spin angular momentum, manifested as circular polarization, is linked to the spins of individual photons, orbital angular momentum, on the other hand, arises from the spatial distribution of the light field itself. Beams of light can be shaped into spirals or helices, with twisted wavefronts. These twisted beams carry orbital angular momentum, characterized by an integer value mℏ per photon, where m can take any integer value, positive or negative. If one thinks of stirred water in a glass with a spoon, there is momentum flow around the axis of the glass. Light can behave just the same. There can be an optical momentum circulating around the axis of the light beam, and this light beam is then said to carry orbital angular momentum. For light beams, we do not have a spoon, but we can introduce this twist by designing a laser cavity, or, more easily, by designing a diffraction grating (i.e., hologram) to do so. We can use the picture below to describe the OAM derived from helical phase fronts where the wavefront of the light beam resembles a screw thread or pasta fusielli. The length/pitch of the screw thread relates to the wavelength of the light, and the rotation rate determines the frequency of the light. This concept of the orbital angular moment of light dates back nearly 100 years. Interestingly, the author of the key paper in the 1930's was Darwin, grandson of Charles Darwin. OAM is linked to higher order, e.g., quadrupole transitions, where the angular momentum based on polarization alone is not enough to drive a transition. The study of the angular momentum of light combines fundamental physics with potential technological applications, offering a richer understanding of light properties and enabling novel practical uses.

**Q2. Could you please highlight some of the important progress in OAM research?**

The development of the OAM of light has seen significant advances from a theoretical concept into a practical tool with numerous applications over the past few decades. The concept of OAM in light beams was formally introduced, distinguishing it from SAM in the early 1990s by Les Allen and coworkers. In the 2000s, techniques to generate and detect OAM beams, such as spiral phase plates, spatial light modulators (SLMs), and q-plates, were developed. These innovations have made experimental investigations feasible. Early applications of optical tweezers and particle manipulation by Halina Rubinsztein-Dunlop and others showcased the practical potential of OAM. OAM became truly quantum in 2001 when Anton Zeilinger and coworkers showed that OAM could be quantum entangled between two photons and that this entanglement was highly dimensional. Experiments have demonstrated the advantages of OAM in multiplexing and secure data transmission.

The OAM is fundamentally interesting in terms of understanding and manipulating the properties of light. In parallel with the application areas, the study of OAM has focused on what it tells us about light. At one level, fields are fields, and one could describe or numerically model their behaviors on any complete basis, e.g., Hermite Gaussian modes or even plane waves. However, in regard to an intuitive understanding of a particular phenomenon, it is often useful to consider a basis that reflects the question. Hence, the OAM lends itself to understanding angles, rotating frames, or circular boundary conditions. These have led to simplified understandings of, e.g., angular uncertainty relationships and rotational frequency shifts.

However, perhaps more important than all of this is that the OAM shows the potential for the spatial structuring of a light beam in terms of its phase and intensity. This is a new degree of freedom by which light beams could be optimized for their intended role.

**Q3. How would you characterize the development of the application aspects of this field over the past few decades? In which areas or aspects does OAM perform better than the spin angular momentum?**

I think the first application of orbital angular momentum that attracted the attention of the optics community was the work of Halina Rubinstzein-Dunlop and coworkers in 1995. They used an OAM-carrying beam to set a microscopic part into rotation (within optical tweezers). The use of OAM beams in microscopy can lead to increased resolution beyond the diffraction limit. It can also improve image contrast by exploiting the different interaction mechanisms of OAM modes with matter.

In the 2010s, while superresolution imaging techniques leveraging OAM were being developed, significant strides were made in the use of OAM to encode quantum information and achieve high-capacity optical communications. OAM allows information encoding in higher-dimensional Hilbert spaces, which is beneficial for quantum cryptography and computing to enhance security. Mehul Malik’s group at Heriot Watt University in Scotland is probably the world leader in the use of OAM for high-dimensional studies. OAM modes can also be used to increase data capacity in optical communications since they enable the multiplexing of multiple data channels on the same frequency. This is crucial for addressing the ever-growing demand for information processing. In 2004, our own work here in Glasgow demonstrated that OAM could be used to increase data transmission rates by encoding information in different angular momentum states. In 2012, Alan Willner and coworkers showed that this could be implemented with Terabit rates. Currently, many groups worldwide use spatial light modulators for classical and quantum beam and photon shaping. These modulators are already available from companies such as Hamamatsu, Meadowlark Optics, or Holoeye.

The difference between OAM and SAM can be seen in this way: While SAM relates to the polarization of light and provides for two states (left and right circular polarization), OAM offers an infinite number of states because of its dependence on the spatial phase structure of the light beam. This means that each photon with OAM has a multiplicity of states to carry more information, making it superior for high-capacity data transmission. The way in which OAM beams interact with materials and structures can be distinctly different from that of SAM, offering unique advantages in specific applications such as microscopy and materials science.

**Q4. Currently, what are the major challenges and where is the fundamental and application research focused on in this area?**

Despite the advancements, several challenges persist in the field of OAM: OAM modes are susceptible to distortions caused by atmospheric turbulence and imperfections in optical components, which can degrade performance in communication systems and imaging applications. In practical systems, maintaining orthogonality and minimizing crosstalk between different OAM modes can be challenging. Scaling up the number of OAM modes for multiplexing while maintaining efficiency and low error rates remains an area of active research. Integrating OAM-based systems with existing fiber optic networks and quantum communication infrastructure poses technical and logistical challenges. Accordingly, current research for OAM addresses these challenges and explores new frontiers: the development of robust methods for generating and detecting OAM modes with higher efficiency and lower error rates. OAM can be combined with other multiplexing techniques (e.g., wavelength-division multiplexing) to further increase the data transmission capacity.

Today, many great groups have explored the applications and understanding of OAM. Given the advances in technology and a deeper understanding of light‒matter interactions, the deployment of OAM-based communication systems, I believe, could lead to a dramatic increase in data rates and enhanced security features, particularly for free-space optical links and satellite communications. With respect to prospects, it is hard to predict how far research could reach in the future. All I can do is express what I am interested in. OAM is an example of the spatial structuring of light. Similarly, Bessel beams, Airy Beams, and many other beam types have been explored.

**Q5. You are a renowned scientist in OAM research. Would you like to tell us what motivated you to initially enter the field of OAM research? Who (and/or what) played important roles?**

When I was young, I loved the game “Cluedo” (or *Clue* as it’s known in some places). I don’t know why, but I always chose to be Professor Plum. Perhaps it was an early hint at my future career, as though I was destined for academia.

When I was an undergraduate, my first choice for a final year project was actually geophysics one. However, that project was oversubscribed, so I was assigned my second choice, which was building a carbon dioxide laser. Although it wasn’t what I had initially intended, it opened my eyes to the world of optics and lasers, which ultimately shaped the path of my research.

When I started my own research as an early-career researcher (ECR), I wrote a grant application on optical parametric oscillators. A representative from the funding body came to discuss it. At dinner that evening, we instead ended up talking about his own research—a recent paper he had published in 1992. I found the topic fascinating. His name was Les Allen. Since then, and until his death 2016, he was a wonderful mentor and collaborator throughout my career. The conversation at that dinner changed my research interest and set the direction for my career forever. I owe him many things (including him introducing me to the Harry Potter books!), and I am always grateful for his invaluable influence on my career. Thank you, Les.

**Figure:**
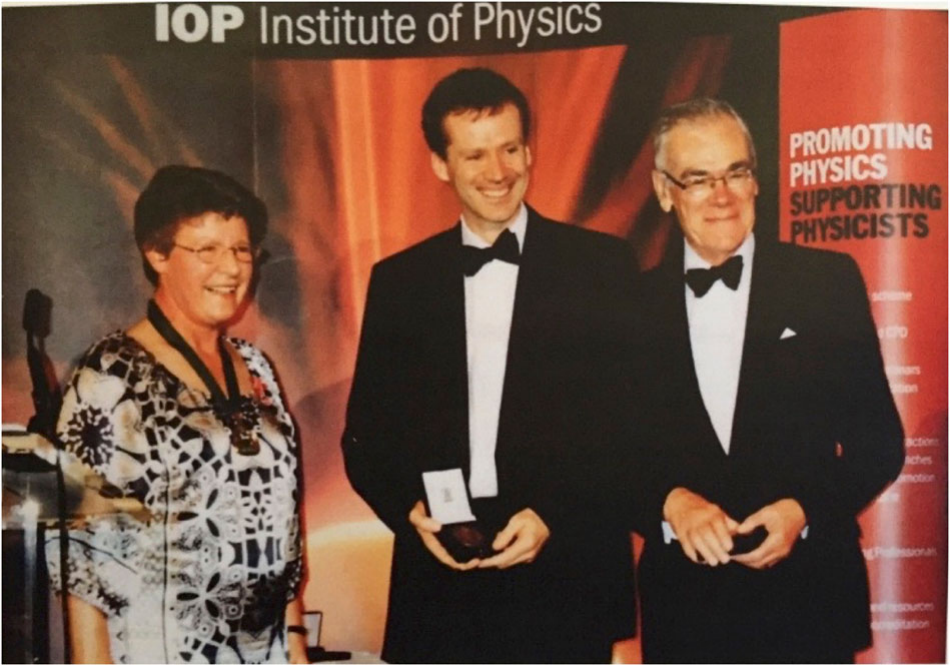
Prof. Miles Padgett received the IOP Young Medal and prize with Prof. Les Allen for their work on OAM

The key to my whole career has been the support of the Royal Society. Their award to me of a University Research Fellowship in the mid-1990s gave me the job security to try something new, something that might not work, but fortunately did! I have been fortunate enough to have them support me throughout my career most recently by the award of a Research Professorship, allowing me to dedicate my time to research and again to try something new.

**Q6. In addition to the area of OAM, your research interest over the years has also covered a wide range of topics involving different properties of light, classical, or quantum. How did you choose a new project or direction of investigation, or were they all initiated from one topic or area that then evolved into another?**

Although it looks like I’ve done many different things—I’m not sure I have. I have always looked for things where I can apply the skills of mine or my research group. For much of what we have done, we have used our skills in shaping light beams, for OAM, for tweezers, for quantum measurement, for imaging, etc. In most projects, I learn new things too. The key is to take these new things, add them to your existing skill set, talk to people, and discover where and how to apply these skills to something new. By talking, listening, being curious, and being interested, I stumble across new ideas or new topics that I find fascinating.

The challenge is to find a way to fund the idea. As researchers, we are embedded in a project culture where projects are designed, funded, executed, and finished, and then their impacts are assessed. My own approach is that this project structure applies not only to research but also domains when one wishes to effect change.

However, often it is not the topic; it’s the people you find to work with that makes you think. I think the secret of success is working with the right people, which very much includes the next generation, e.g., your research students. Their fresh ideas, enthusiasm, and problem-solving abilities pushed me to think creatively and approach problems from new angles. Many breakthroughs were made through these collaborations. I’m still learning how to find the right people or let them find me—but recognizing that the team you are part of is more important than where the idea starts.

**Q7. Apart from fundamental research, have you ever worked with industry partners for further development of your research outcome? If so, how did you perceive the needs of the companies? What are the achievements thus far?**

Yes, I often worked with industry partners, although rarely with OAM directly. At different times in my career, I have worked with both large companies such as Siemens and Shell but often with smaller companies too. I also work with government departments. These industry partners are crucial for gaining insights into real-world applications and challenges. They often trust our academic expertise to push boundaries, and that trust is vital. These relationships have helped me stay connected to practical, impactful uses of our work, and I also value the intellectual diversity and perspectives they bring. My industrial work has been mainly in response to their articulated needs rather than the concept push. Some researchers work best by simply thinking. However, I find that I work best when listening to others and then reflecting on how my knowledge and skills can contribute or how I might now think differently about what I once believed I understood. Some of the industrial projects that my group supported included the construction of a Fourier transform spectrometer with no traditional mechanical parts, holographic optical tweezers, and a gas sniffing system for identifying oil reserves. I loved working on all these projects, not only because of the challenges but also because of the skills I gained, and, most importantly, the people I had the chance to work with.

Optical tweezers were originally invented by Arthur Ashkin in 1986. He received the Nobel Prize in 2018 for this invention. I met Arthur several times. He was a great scientist, but even more importantly a lovely person—very supportive of the next generation of researchers. Our holographic optical tweezers were an extension of the original optical tweezer concept. Optical tweezers can be used to manipulate microscopic particles with light. This is delicate work, requiring precision and control. The key was developing algorithms that could be utilized to dynamically shape light fields using computer-generated holograms. This project required synergy between optics and computational techniques. The gas sniffing system presented an exciting opportunity to apply our expertise in a completely new context. It allowed us to collaborate with industry partners in the energy sector, which brought additional learning and practical impact—not least an introduction to solving inverse problems.

Of course, collaborating with industry partners provided funding that supported our research by paying for students and reinvesting in lab equipment. In addition, I’m a keen cyclist. My first proper racing bike was bought with some money that I received from licensing an idea to industry.

**Figure:**
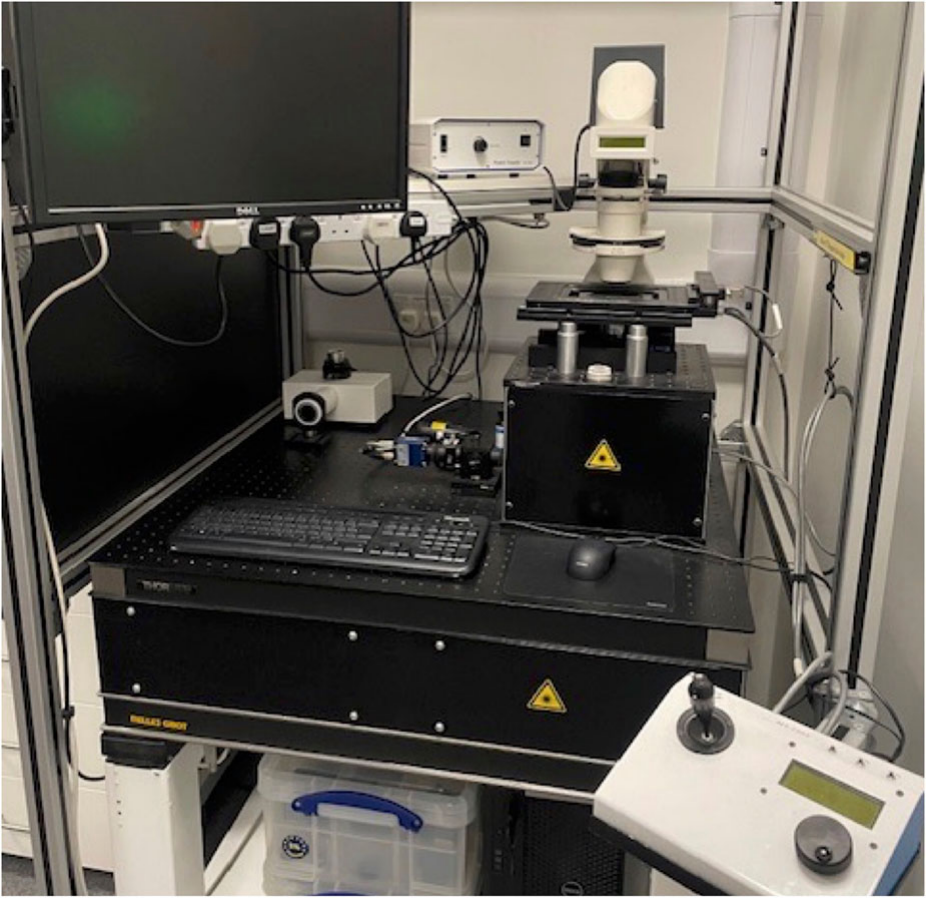
Holographic tweezers built by Graham Gibson in my group

**Q8. You were the principal investigator (PI) for QuantIC. Could you please tell us about QuantIC and give some examples of what your team is currently working on?**

QuantIC was one of the UK’s four quantum technology hubs. It focuses on imaging technology. I was the lead scientist of QuantIC. The Hub is funded by the UK Government’s funding agency: the Engineering and Physical Sciences Research Council. It coordinates quantum imaging research across 8 universities and works closely with 50 UK-based companies. Within QuantIC, more widely, my colleagues have developed new types of quantum-enhanced multidimensional cameras with breakthrough functionality that can see in 3D or measure fluorescence lifetimes, sources of entangled light, and cameras that can be used to detect greenhouse gases. In the next phase of the UK National Programme (2024-2029), we have created a new hub, QuSIT, with Mike Holynski in Birmingham which combines both photons and atoms and applies them to both sensors and imaging.

Throughout my career, one thing has kept coming back—namely, the ability to shape light beams in terms of both their intensity and phase—OAM is just one example, albeit the first. One of my current research interests is to create a new type of endoscope. Traditional endoscopes use bundles of optical fibers, one fiber for every image pixel, to relay an image from one point to another. Inspired by the excellent work of Tomáš Čižmár and coworkers, we have been working on an endoscope based on a single optical fiber that is the width of a human hair. The key to this approach relies upon the complicated beam shaping of a laser at the entrance of the fiber such that it creates a scanning spot at the output. We now glue our fiber inside a syringe needle, we are targeting medical uses where the needle will ensure minimally invasive imaging.

**Figure:**
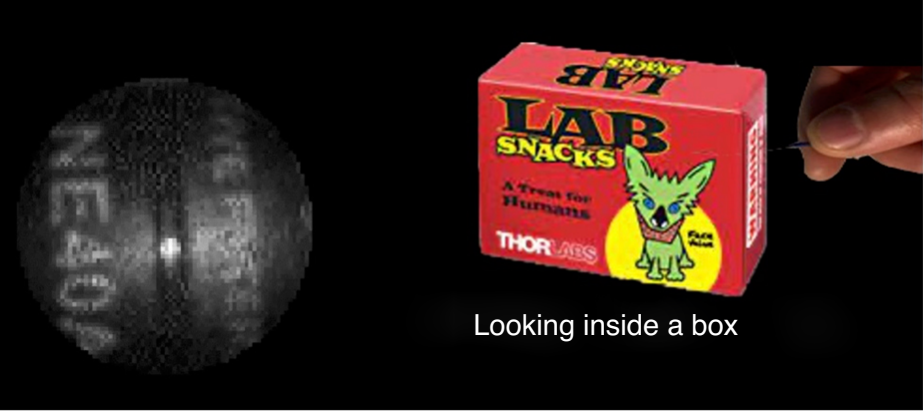
Endoscope based on a single fiber embedded in a syringe needle looking inside a box

**Q9. You are not only a successful scientist but also an academic administrator, such as Vice Principal (VP-r) for research at Glasgow University. Your experience as a VP-r is unique among LSA interviewees. What are the efficient approaches for universities to support and encourage fundamental research and innovation?**

I spent five years as Vice Principal for Research. My goal was to improve the research at Glasgow. My primary ambition was to develop Glasgow University in terms of both fundamental and applied research to increase the University’s visibility on the global stage. I aimed to ensure that our academic community was not only conducting cutting-edge research but also translating that research into real-world impact. This involved improving our infrastructure, securing more external funding, and building strong partnerships across academia, industry, and government.

My first act as VP-r was to take advantage of my network of world-leading researchers in different disciplines. I invited them to visit Glasgow, speak to each department in turn, and articulate what world-leading research looked like. I was clear that we were not doing this for an assessment but to inform and inspire. The second thing I did was to create an environment that fostered internal and external interdisciplinary collaboration and secured funding from both industry and government sources. As a result, the research Glasgow published moved from being assessed as being in the lower quartile among the UK’s largest and oldest universities in 2014 to being in the upper quartile in 2021. I am proud of what we achieved.

When working, as the Interim Executive Chair for EPSRC in 2023, I successfully led an initiative to increase the number of funded Centres for Doctoral Training by nearly 50%, which represented an additional 1500 students, which is one of my proudest achievements in my career to date.

**Q10. You have worked for many research committees/advisory boards, such as the Engineering and Physical Sciences Research Council (EPSRC) and the International Society for Optics and Photonics (SPIE). What is the motivation for you to take these roles? How does this benefit you or your research?**

I have been lucky to do different things at different stages of my career. When I was younger, like many of us, I complained about the way some things were done. However, I also believe that if we want to complain, we have to be prepared to step up. Serving on research committees and advisory boards allows me to advocate for areas of science that I believe hold great promise for advancing human knowledge and solving global challenges. My role has a direct impact on the direction of science and research policy. In this way, I hope to contribute to ensuring that funding and support are allocated fairly to areas that have the potential for significant scientific and societal impact.

While contributing to society, I certainly benefit from these engagements as well. Engaging with a wider community of researchers, policy-makers, and industry leaders provides broader insights into the scientific landscape. I learn from others’ perspectives, and this, in turn, enriches my own research and understanding of where my work fits into the larger picture. Serving on these boards also builds relationships with key figures in the academic, government, and industry sectors. These connections often lead to fruitful collaborations that benefit not only my own work but also the wider academic community.

**Q11. When undertaking several roles simultaneously, how do you balance your time for research, committee service, and academic administration?**

When I was Vice Principal for Research at Glasgow University. My contract involved working 50% as a VP and 50% as a researcher. I’ve always been clear that I wanted to continue to be a researcher, to work alongside and mentor the next generation of researchers. I think having enough time is all about focusing on the small number of things that truly make a difference. In leadership roles, I need to create bandwidth for myself and my teams to deliver effectively. This involves not only deciding what to pursue but also being willing to say no. I was strict with my time and careful not to waste the time of my teams either.

I’ve been lucky to work with great teams both in my research and my other roles; ultimately, whether it be other researchers or professional services it is the team that succeeds not the individual.

**Q12. Do you have to work overtime? If so, does it disturb your family life?**

Yes, sometimes I work during the evening and weekends. However, I must say that we are lucky in that the government pays us a salary to do something we love; my work is also my hobby. Yes, we all work hard—but it seems the least we can do is to repay the privilege we have.

As for my personal life, I am incredibly fortunate to have a supportive and accomplished partner. My wife, Heather Reid, was formerly a well-known weather presenter for BBC Scotland. She was awarded an OBE (Order of the British Empire) and now works in various capacities related to the support of nature and our natural environment. We are both successful in different walks of life. We understand, respect, and support each other’s careers.

**Q13. You have won various national and international prizes/awards (Young Medal, Lord Kelvin Medal, Max Born Award, FRS, etc.) for your research. As a scientist with many spectacular achievements, what advice would you like to give to junior scientists who are in the early stages of their careers?**

Honestly, beyond having generous supporters, luck also plays a large part in winning prizes. I have been lucky throughout my career. However, if you never flip the coin, it will never come up heads. You must continue with innovative research and show some novel work. As I said previously, my research is kind of my hobby. No matter if I win the prize or not, I enjoy doing it. As a scientist, I’m not rich—but wealthy enough to be okay to concentrate on my research to explore an unknown world, satisfy my curiosity and impact society as well. I recognize that academic success is a bit of a lottery—whether it is the grant application that has just been approved, the favorable referee reports, or the project that worked better than you could ever have hoped for. Sometimes, others appreciate what you have done, and things fall into place; other times, they don’t, and you have to try again. Resilience is essential.

Although I’ve been lucky at times, there have been lots of times when I find that I chose the wrong project—the one I tried but later realized wasn’t feasible. In such cases, it is important to give up and move on. You need to forget what has already been invested and focus on whether or not continuing is worth the effort for the future. It’s also crucial to remember that you are not only investing your own time but invariably the time of others as well.

**Q14. Do you have any other hobbies apart from your research work? What are the things or activities you enjoy when not at work?**

I have my hobbies. When I was a younger person, I used to rock climb and ski a lot. Some people think these are dangerous sports. However, unless you are truly pushing the envelope, which I’m not, then it is manageable—rock climbing is one of those things that feels a lot more dangerous than it is! What is great about it is that when you are there on the high end of the rope, nothing else matters; your whole world becomes the one meter around you—time goes quickly or slowly, it just depends on the climb, but it becomes totally absorbing. More recently, I have cycled both on and off the road. I like building bicycles and then riding them with my friends whenever I have time. My latest toy is a gravel bike that somehow catches the best aspects of both on and off-road cycling, offering me the freedom to explore varied terrain. I find that maintaining a healthy balance between career, family, and personal interests is crucial for long-term success and happiness. Cycling gives me a mental and physical outlet and helps me recharge and return to my work with renewed energy and perspective.

**Figure:**
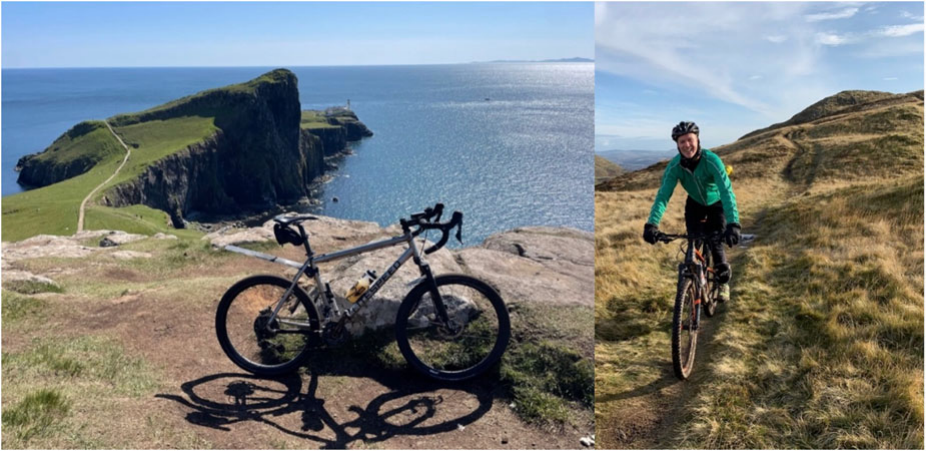
Miles builds bicycles and enjoys riding the bicycles

